# Liver Transcriptome Analysis Revealed the Physiological Response Mechanism of Red-Spotted Grouper (*Epinephelus akaara*) Under Acute Hypoxic Stress

**DOI:** 10.3390/ani16101469

**Published:** 2026-05-10

**Authors:** Yukun Huang, Qiaoyi Chen, Xueqin Hu, Zhiya Yu, Xiyin Zheng, Jinhui Wu, Tianguang Cai, Yuhua Cui, Along Gao, Hu Shu

**Affiliations:** 1 School of Life Sciences, Guangzhou University, Guangzhou 510006, China; 18718615843@163.com (Y.H.); chenqyee2000@163.com (Q.C.); huxueqqq@163.com (X.H.); 13418837975@163.com (Z.Y.); zhengxyinnn@163.com (X.Z.); 2 Agro-Tech Extension Center of Guangdong Province, Guangzhou 510520, China; wjhin@sina.com; 3 Shenzhen Haijuyuan Aquacture Technology Co., Ltd., Shenzhen 518120, China; caizong2026@163.com (T.C.); czong2026@163.com (Y.C.)

**Keywords:** *Epinephelus akaara*, hypoxia, RNA-seq, liver

## Abstract

*Epinephelus akaara* is one of the aquaculture species with high commercial value in the aquaculture industry in the Asia-Pacific region. However, acute hypoxia is widespread in the intensive farming and live fish transportation of *E. akaara*, which seriously hinders the rapid development of the *E. akaara* farming industry. In this study, transcriptome sequencing (RNA-seq) was conducted on the liver tissues of *E. akaara* after hypoxia (0.6 ± 0.1 mg/L) for 1, 3, 6 and 9 h. Kyoto Encyclopedia of Genes and Genomes (KEGG) enrichment analysis revealed that key signaling pathways such as IL-17, HIF-1, TNF and PI3K/Akt were significantly enriched in the hypoxia treatment groups (Hy6 and Hy9). Reverse transcription quantitative polymerase chain reaction (RT-qPCR) analyses revealed that genes linked to energy metabolism, immune response and apoptosis changed significantly in the liver and brain at 6 h and 9 h under acute hypoxia. In addition, three metabolism-related enzymes (GLU, LDH and PK) and two immune-related enzymes (ALT and AKP) all changed significantly at 6 h and 9 h, indicating that 6–9 h was the critical period for physiological stress of *E. akaara*. In conclusion, these results indicate that *E. akaara* mediated the organism’s tolerance and adaptation to hypoxic stress by regulating energy metabolism, immune response and apoptosis. This study enriches the regulatory mechanism by which *E. akaara* adapts to acute hypoxia and is conducive to the breeding of hypoxia-tolerant *E. akaara* strains.

## Simple Summary

*Epinephelus akaara* is a highly valuable aquaculture species in the Asia-Pacific region. However, intensive farming and live transport often expose it to low oxygen conditions, resulting in substantial economic losses. This study investigated the hypoxic response of *Epinephelus akaara* by analyzing hepatic gene expression and enzymatic activities following 9 h of hypoxia exposure. The results demonstrated that acute hypoxia at 6–9 h induced significant alterations in genes associated with energy metabolism, immune response, and apoptosis, with activation of key signaling pathways including HIF-1, IL-17, and PI3K/Akt. Enzyme activities related to energy production and immune function also exhibited significant changes during this critical period. These findings reveal that 6–9 h is a critical window period for physiological stress and adaptation to hypoxia in *E. akaara*. These results can support the development of hypoxia tolerant varieties to improve the sustainability of grouper farming and reduce losses.

## 1. Introduction

In the field of aquaculture, water quality is a complex system integrating a variety of physical, biological and chemical factors, and the quality of water is directly related to the growth and development of cultured fish [1]. Although the aquatic environment for fish culture is influenced by a multitude of water quality parameters, including temperature, dissolved oxygen, carbon dioxide, ammonia, nitrite, and pH, only a select few of these key factors play a decisive role in the growth and development of cultured fish [2]. Among these, dissolved oxygen (DO) constitutes a particularly critical limiting factor, as the aerobic metabolism of fish is fundamentally dependent upon maintaining adequate DO levels [3]. In aquatic ecosystems, hypoxia in fish is defined as a condition in which dissolved oxygen levels fall below the threshold required to support normal physiological and metabolic functions, typically characterized by DO concentrations below 2 mg/L [4,5]. The phenomenon of hypoxia is the result of multiple factors such as seasonal variation, circadian rhythm, water temperature, salinity, water stratification, surface runoff, water eutrophication, intensive aquaculture, global warming, water pollution and so on. These factors affect the generation, consumption and distribution of dissolved oxygen, resulting in the decline of water dissolved oxygen level. In turn, it negatively affects the survival of aquatic organisms and ecosystem health [6,7]. In addition, as the market demand for fresh aquatic products continues to grow, the aquaculture industry is rapidly developing towards intensive production and high-density live transportation. However, it also increases the frequency of hypoxia in aquaculture systems and affects the stability of the aquatic supply chain, resulting in significant economic losses [8,9]. Consequently, the molecular mechanisms underlying the fish response to acute hypoxia have attracted growing research interest in recent years.

Hypoxic stress adversely affects various physiological processes in cultured fish, including growth and development rates, hatching delays, feed efficiency, metabolism, and feeding capacity [10,11,12]. Consequently, to maximize oxygen uptake from the environment and sustain normal metabolic functions, fish have evolved a range of adaptive strategies for hypoxia tolerance. Notable examples include gill remodeling in blunt snout bream (*Megalobrama amblycephala*) to increase functional surface area [13], enhanced movement of pectoral fins and body coupled with buccal pumping in zebrafish larvae [14], and rapid circular swimming with mouth agape in yellowtail kingfish (*Seriola lalandi*) under hypoxic conditions [15]. In addition, teleost fish modulate their metabolic strategy to reduce energy consumption by suppressing aerobic metabolic activity and enhancing anaerobic function in response to hypoxia [16]. Under the condition of sufficient oxygen, mitochondria can efficiently synthesize adenosine triphosphate (ATP) through oxidative phosphorylation through the electron transport chain to meet energy demand. However, under hypoxia, due to the limitation of oxidative phosphorylation, cells will turn to anaerobic metabolism to maintain energy supply [17].

The liver has unique functional properties, including nutrient metabolism, detoxification, immune function, mineral and vitamin storage, and synthesis of essential serum components, which affect the physiological processes of the liver, including its oxygen homeostasis [18,19]. The liver of fish and other vertebrates is also regarded as a target organ for a variety of biological and environmental factors. For example, toxins, pollutants, hypoxia, parasites, and other factors can alter the structure and metabolism of the liver [20,21]. Hypoxia causes extensive DNA damage and inflammation in fish hepatocytes, accompanied by the accumulation of hypoxia-inducible factor-1 alpha (HIF-1α) [22,23]. The oxygen-sensitive HIF-1α subunit and the constitutively expressed hypoxia-inducible factor-1 beta (HIF-1β) subunit jointly constitute the transcription factor HIF-1 [24]. HIF-1 is known to be a master regulator of hypoxia-induced gene expression in fish and mammals, enhancing the expression of a variety of hypoxia-induced genes, including vascular endothelial growth factor (*vegf*), erythropoietin (*epo*), genes involved in glycolysis and metabolism (e.g., lactate dehydrogenase, *ldh*) [25,26]. Through a variety of regulatory mechanisms, HIF-1 enables hepatocytes to rapidly respond to changes in oxygenation levels in the environment [27]. HIF-1 binds to the hypoxia response element (HRE) in the *vegf* promoter to induce the activation of *vegf* transcription, and acts as an effective activator of angiogenesis [28].

*Epinephelus akaara* is a species of considerable economic importance within the grouper genus, native to the coastal waters of Southeast and East Asia [29,30]. Owing to its exceptional market acceptance and stable consumer demand, *E. akaara* has emerged in recent years as a key commercial species for the aquaculture industry in the Asia-Pacific region [31]. RNA-seq is generally regarded as a reliable sequencing technology for studying non-model organisms and has greatly promoted the study of the molecular mechanisms of the acute hypoxia response in fish at the genomic level [32]. Utilizing RNA-seq, this study analyzed the response of *E. akaara* to acute hypoxia and identified differentially expressed genes (DEGs) between hypoxic and normoxic groups. In addition, this study utilized RT-qPCR to validate hypoxia-responsive candidate genes and performed quantitative analysis of the expression profiles of key hypoxia-related genes in the liver and brain. By integrating gene expression levels with enzyme activity assays, the regulatory effects of energy metabolism, immune response, and apoptosis in the hypoxia adaptation of *E. akaara* were further revealed. The results of this study provide a new perspective for understanding the mechanisms of hypoxia adaptation in grouper, and provide new ideas for the development of molecular breeding techniques for new strains tolerant to hypoxia.

## 2. Materials and Methods

### 2.1. Ethics Statement

The animal study was approved by the handling procedures and experimental protocols of all animals in this experiment were in accordance with the Experimental Animal Ethics Committee of Guangzhou University of China. The study was conducted in accordance with the local legislation and institutional requirements.

### 2.2. Acute Hypoxic Exposure and Sample Collection

The healthy grouper (average standard length: 17.1 ± 0.9 cm, average standard weight 77.9 ± 13.5 g) used in this study were provided by Guangdong Marine Fishery Experimental Center. Before the experiment, *E. akaara* were acclimated for 2 weeks in a cylindrical tank containing a recirculating seawater system with routine disinfection. The salinity was maintained at 20–30‰, and the water temperature was controlled at 30 ± 1 °C. During the acclimatization period, the fish were fed with a diet twice a day at 3% of their body weight. A total of 120 healthy fish were selected and fasted for 24 h before being evenly distributed into three tanks (as parallel groups), with 40 fish per tank. At the beginning of the experiment, DO was reduced from 6.0 ± 0.1 mg/L to 0.6 ± 0.1 mg/L within 1 h by reducing the water volume to one-third of the barrel volume, stopping aeration, cutting off the circulating water supply, and covering the plastic film, which was recorded as Hy0 [33,34,35]. During the whole experiment, DO was maintained at 0.6 ± 0.1 mg/L by adjusting oxygen supply and water volume, and the change in DO was monitored by a dissolved oxygen detector (Leici, Shanghai, China). Under the condition of maintaining the dissolved oxygen level, three fish were randomly selected from each barrel at 0, 1, 3, 6 and 9 h, respectively, which were set as the control group Hy0 and the hypoxia experimental groups Hy1, Hy3, Hy6 and Hy9 [5]. Following anesthesia, the fish were dissected to collect liver tissues. Tissues intended for RNA analysis were immediately placed in centrifuge tubes containing RNA Keeper Tissue Stabilizer (Vazyme, Nanjing, China). Those designated for enzyme activity assays were washed with PBS, transferred into empty centrifuge tubes, and promptly stored in liquid nitrogen.

### 2.3. RNA Extraction, cDNA Library Construction and Sequencing

Total RNA was extracted using Trizol reagent (Thermo Fisher Scientific, cat.15596018, Waltham, MA, USA) in strict accordance with the operating procedure provided by the manufacturer. RNA concentration and purity were assessed by using the Bioanalyzer 2100 system and RNA 6000 Nano LabChip Kit (Agilent, San Jose, CA, USA, 5067-1511). High-quality samples with RNA integrity index (RIN) greater than 7.0 were selected for sequencing library construction. The cleaved RNA fragments were reverse transcribed into cDNA using SuperScript™ II reverse transcriptase (Invitrogen, cat.1896649, Thermo Fisher Scientific, Waltham, MA, USA), and this was used as a template for the synthesis of U-labeled second strand DNA. The U-labeled second strand DNA was treated with UDG enzyme (NEB, cat.m0280, Ipswich, MA, USA) and ligated products were amplified by PCR under the following conditions: initial denaturation at 95 °C for 3 min, eight cycles of denaturation at 98 °C for 15 s, annealing at 60 °C for 15 s, and extension at 72 °C for 30 s were performed. The final extension was at 72 °C for 5 min. The average insert size of the final cDNA library was 300 ± 50 bp. Three biological replicates were used in each group. Finally, 2 × 150 bp paired-end sequencing (PE150) was performed according to the standard operating procedure of the Illumina Novaseq™ 6000 platform, Illumina, San Diego, CA, USA.

### 2.4. Sequence Concatenation and Functional Annotation

Raw reads were filtered using Cutadapt (v1.9) to remove reads containing adaptor sequences, polyA/polyG sequences, unknown base (N) ratio more than 5%, and low-quality base (Q value ≤ 20) ratio more than 20%. After filtering, FastQC (v0.11.9) was used to evaluate the quality of clean reads, including Q20, Q30 and GC content. Clean reads were aligned to *E. akaara* reference genome using HISAT2 (v2.2.1), and alignment verification was performed based on a database of potential splice sites. The aligned reads were assembled into transcripts using StringTie (v2.1.6), and gffcompare (v0.9.8) was used to integrate the transcripts of all samples to construct a comprehensive transcriptome. Finally, StringTie and ballgown were used to calculate gene expression, and the expression level of mRNA was expressed as FPKM (number of alignment fragments per kilobase transcript per million) value, and functional annotation analysis was performed.

### 2.5. Identification and Enrichment Analysis of Differentially Expressed Genes (DEGs)

DEGs analysis was performed by DESeq2 software Version 1.26.0 for comparison between groups. The screening conditions were genes with false discovery rate (FDR) < 0.05 and absolute log_2_ fold change |log_2_FC| > 1. Gene Ontology (GO) function enrichment analysis and Kyoto Encyclopedia of Genes and Genomes (KEGG) pathway enrichment analysis were performed on the selected DEGs. To comprehensively reveal the key molecular regulatory networks and biological functions in hypoxic adaptation, the KEGG pathway categories of metabolism, cellular process, environmental information processing, human disease and genetic information processing were screened for further analysis.

### 2.6. RT-qPCR Validation

In order to verify the reliability of RNA-seq data, 24 DEGs were selected for RT-qPCR analysis. Based on the mRNA sequence of *E. akaara*, the specific primers for DEGs were designed by Primer v.6.0 software (Table 1), and *actin beta* (*β-actin*) and *eukaryotic translation elongation factor 1 beta* (*ef-1β*) were selected as the internal reference genes for expression standardization. RT-qPCR was performed on a LightCycler^®^ 480 Instrument II (Roche, Switzerland) by using a 96-well plate (Monad, Suzhou, China). The total reaction system was 20 μL, including 10 μL SYBR qPCR Master Mixture (Yeasen, Shanghai, China), 3 μL RNase-free ddH_2_O, 2 µL each of forward and reverse primers at a concentration of 10 µM, 3 μL cDNA (concentration 10 ng/μL). The RT-qPCR reaction conditions were as follows: predenaturation at 95 °C for 30 s, followed by 40 cycles of 95 °C for 10 s and 60 °C for 30 s. Melting curves were generated according to the instrument default program: 95 °C for 15 s, 60 °C for 60 s, and a gradual increase from 60 °C to 95 °C at a ramp rate of 0.2 °C/s, during which fluorescence signals were continuously acquired to detect nonspecific amplification and primer dimers [33]. Relative gene expression was calculated by the 2^−ΔΔCT^ method, and quantitative analysis was performed based on CT values [5]. Three sets of biological replicates and three sets of technical replicates were used to ensure data reliability.

### 2.7. Enzyme Activity Detection

The liver tissue used for enzyme activity detection was pretreated according to the instructions of the kit: it was mixed with an appropriate amount of PBS and finally prepared as a 1% tissue homogenate. Three parallel samples were set for each time point, and each parallel sample was used for three replicate experiments. Glucose (GLU: A154-1-1), lactate dehydrogenase (LDH: A020-2-2), alkaline phosphatase (AKP: A059-2-2), alanine aminotransferase (ALT: C009-2-1) were completed on the 96-well cell plate and were detected by multifunctional enzyme labeling instrument (Infinite^®^ 200PRO, Switzerland). Total protein (TP: A045-2-2) and pyruvate kinase (PK: A076-1-1) were detected by UV-visible spectrophotometer (UV1810; Shanghai, China). All kits were obtained from Nanjing Jiancheng Bioengineering Institute (Nanjing, China).

### 2.8. Statistical Analysis

SPSS Statistics 26 software was used to perform one-way analysis of variance (ANOVA) and multiple post hoc comparison on the relative expression levels of target genes. The *t*-test was selected as the calculation method, with a significance threshold of *p* < 0.05 applied to datasets with homogeneity of variance. GraphPad Prism 9.5 software was employed for graphing, and asterisks (*) were used to indicate statistically significant differences in the figures.

## 3. Results

### 3.1. Overview of RNA-Seq Data

RNA-seq analysis was performed on the liver tissue of *E. akaara*. After data filtering, a total of 594,580,620 raw reads (89.18 GB) were obtained, of which 575,478,010 reads passed the quality control process. The clean reads of each library ranged from 4.89 GB to 6.37 GB. The clean reads were compared with the reference genome, the statistical parameters of clean reads were as follows: Q20 was 99.41–99.55%, Q30 was 96.15–97.02%, and GC content was 48.50–54.50%. The clean reads of each library were aligned with the reference genome information of Epinephelus spp., in turn, and the total alignment rate of each sample was 90.68% to 94.01% (Table 2). The RNA-seq raw reads data presented in the study are deposited in the NCBI repository, accession number PRJNA1417131.

### 3.2. Summary of DEGs Between Groups

**Figure 1:**
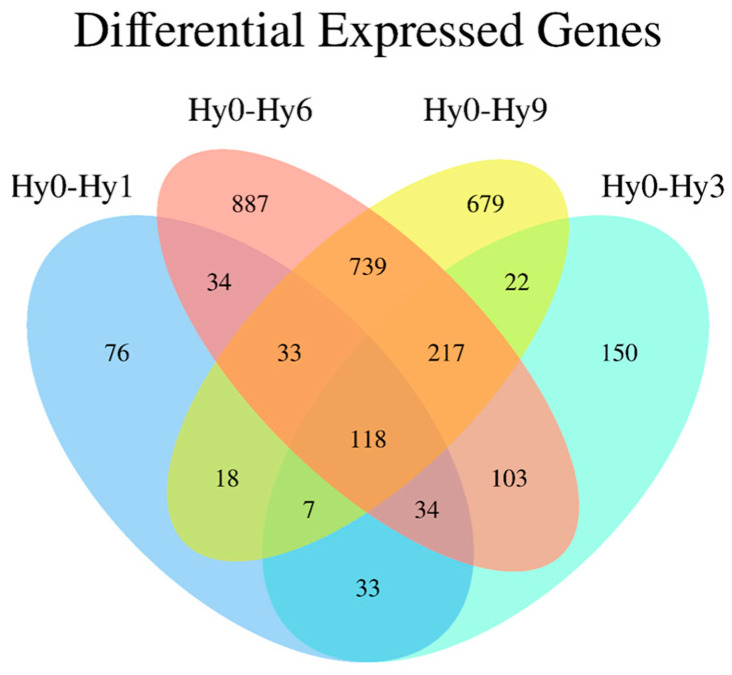
Venn diagram of DEGs in the four comparisons.

**Figure 2:**
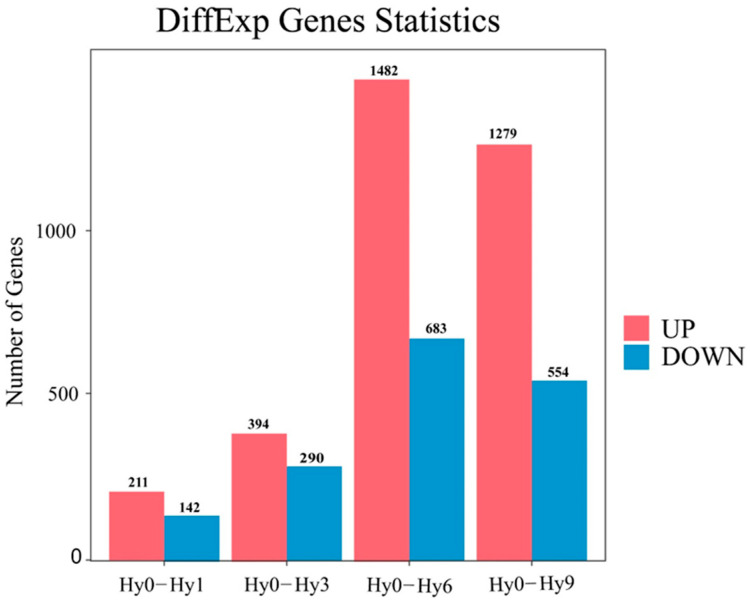
Statistics of DEGs among the four comparisons.

**Figure 3:**
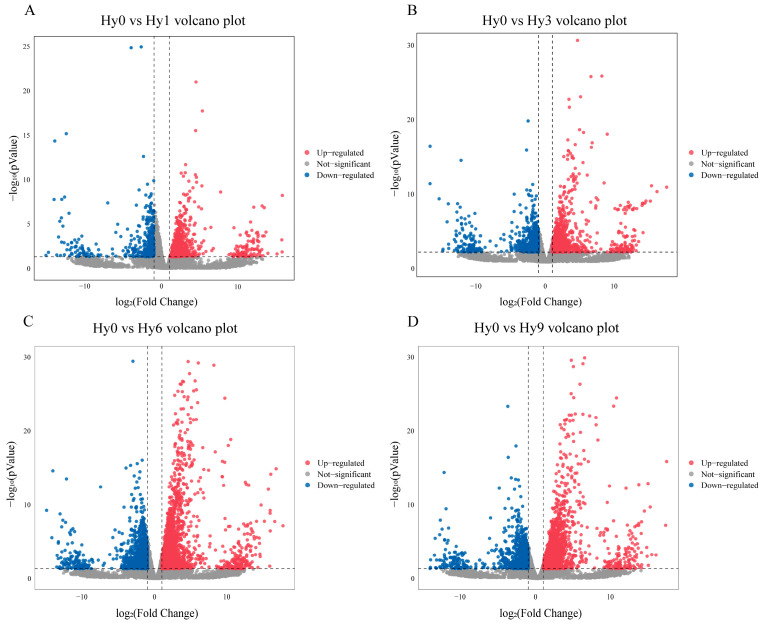
Volcano diagram of DEGs in the liver of *E. akaara* under acute hypoxia, volcano diagram of Hy0 vs. Hy1 (**A**), volcano diagram of Hy0 vs. Hy3 (**B**), volcano diagram of Hy0 vs. Hy6 (**C**), and volcano diagram of Hy0 vs. Hy9 (**D**).

A total of 3150 DEGs were identified between the control group Hy0 and the hypoxic experimental groups (Hy1, Hy3, Hy6, Hy9) (Figure 1). Comparative analysis between groups revealed that there were 353 DEGs between Hy0 and Hy1, among which 211 genes were up-regulated and 142 genes were down-regulated (Figure 2 and Figure 3A), 684 DEGs were found between Hy0 and Hy3, with 394 genes up-regulated and 290 genes down-regulated (Figure 2 and Figure 3B), 2165 DEGs were detected between Hy0 and Hy6, including 1482 up-regulated genes and 683 down-regulated genes (Figure 2 and Figure 3C), Additionally, 1833 DEGs were identified between Hy0 and Hy9, with 1279 genes up-regulated and 554 genes down-regulated (Figure 2 and Figure 3D).

### 3.3. GO Enrichment Analysis of DEGs

**Figure 4:**
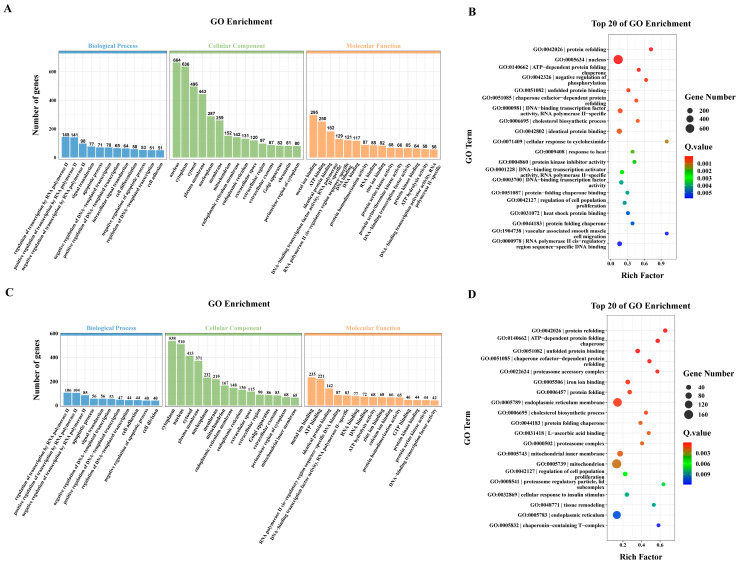
GO enrichment analysis of DEGs in the liver of *E. akaara* under acute hypoxic stress. GO enrichment analysis of DEGs in Hy0 vs. Hy6 (**A**) and Hy0 vs. Hy9 (**C**). The top 20 pathways in GO enrichment analysis of DEGs in Hy0 vs. Hy6 (**B**) and Hy0 vs. Hy9 (**D**). The color of each point represents the adjusted *p*-value of the corresponding entry.

Compared with the Hy0 group, a total of 2165 DEGs were identified in the Hy6 group. The functional annotation analysis showed that these genes were mainly enriched in the following functional categories: In the biological process (BP), including RNA polymerase II regulation of transcription, regulation of DNA template transcription, signal transduction and apoptotic process. In the cellular component (CC) it involved the nucleus, cytoplasm and cell membrane. In molecular function (MF), it mainly includes substance binding and catalytic activity (Figure 4A). The top 20 enriched items in the Hy6 group were mainly involved in protein binding process, including unfolded protein binding (GO:0051082), identical protein binding (GO:0042802), protein-folding chaperone binding (GO:0051087) and heat shock protein binding (GO:0031072). In addition, DEGs were also involved in transcription processes, such as DNA-binding transcription factor activity (GO:0003700), RNA polymerase II cis-regulatory region sequence-specific DNA binding (GO:0000978) and DNA-binding transcription activator activity, RNA polymerase II-specific (GO:0001228) (Figure 4B).

A total of 1833 DEGs were identified in the comparison between the Hy0 group and the Hy9 group, and these genes were significantly enriched in the following functional categories: in biological processes (BP), mainly involved in transcriptional regulation, signaling and cell development; cellular component (CC), covering the cytoplasm, nucleus, and cell membrane; in terms of molecular function (MF), binding activity and catalysis were dominant (Figure 4C). The top 20 gene enrichment terms in the Hy9 group were mainly involved in protein folding and binding processes. These include protein folding (GO:0006457), protein refolding (GO:0042026), chaperone cofactor-dependent protein refolding (GO:0051085), protein folding chaperone (GO:0044183) and unfolded protein binding (GO:0051082). In addition, DEGs were also involved in a variety of biological processes, such as the regulation of cell population proliferation (GO:0042127), cellular response to insulin stimulation (GO:0032869), tissue remodeling (GO:0048771) and cholesterol biosynthetic process (GO:0006695) (Figure 4D).

### 3.4. KEGG Enrichment Analysis of DEGs

**Figure 5:**
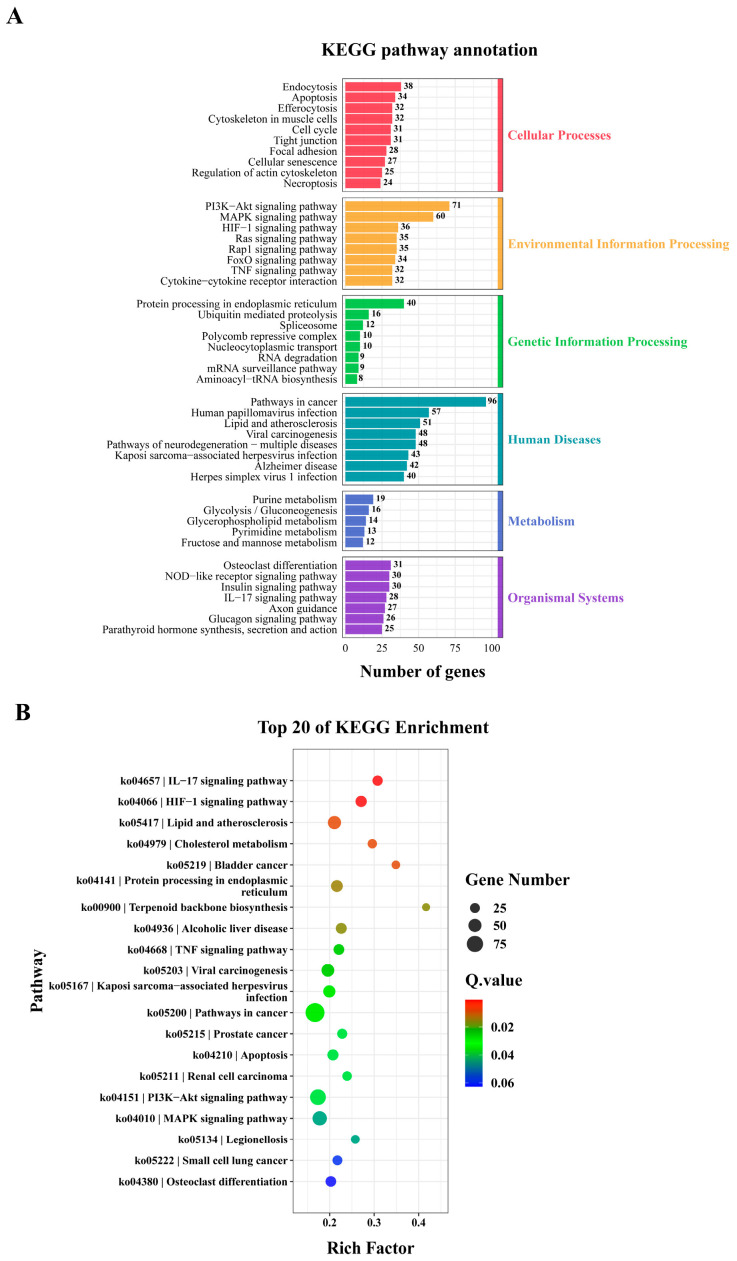

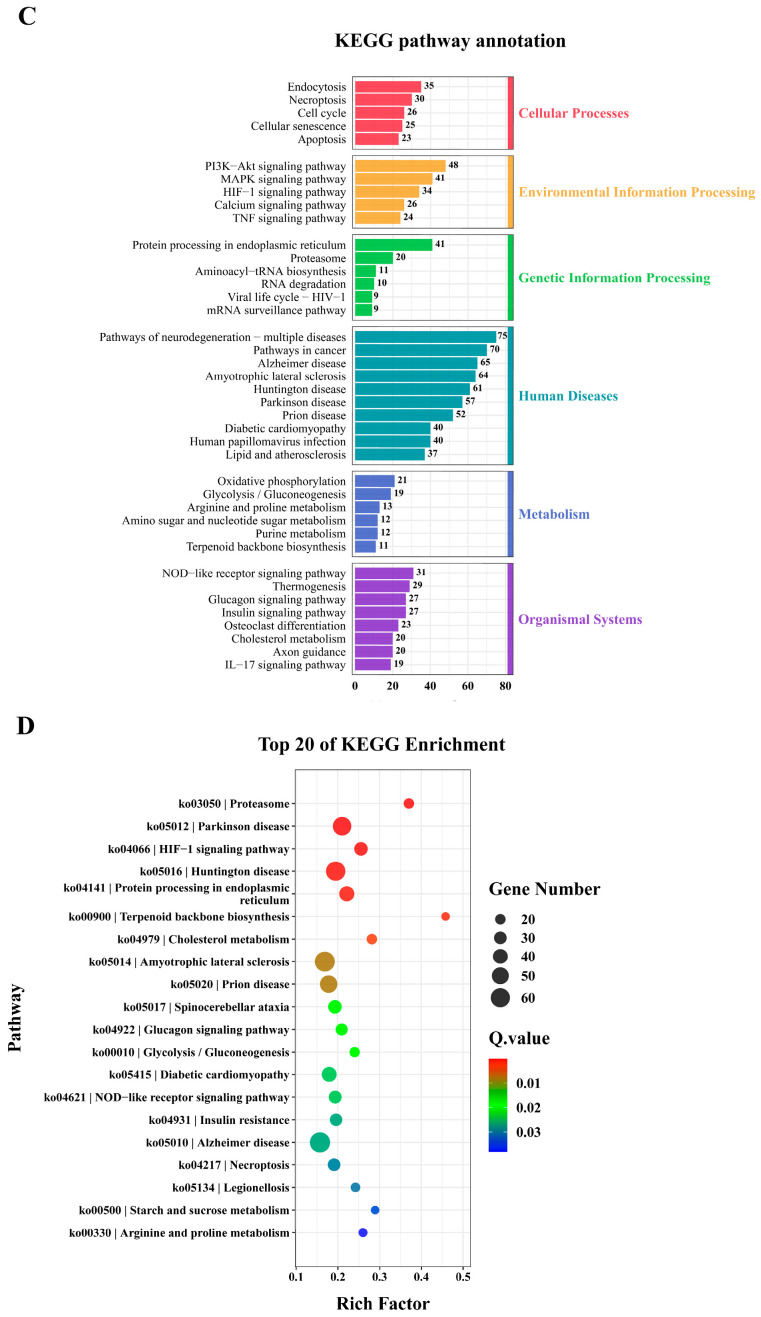
KEGG enrichment analysis of DEGs in the liver of *E. akaara* under acute hypoxic stress. KEGG enrichment analysis of DEGs in Hy0 vs. Hy6 (**A**) and Hy0 vs. Hy9 (**C**). The top 20 pathways in KEGG enrichment analysis of DEGs in Hy0 vs. Hy6 (**B**) and Hy0 vs. Hy9 (**D**).

KEGG pathway analysis showed that 1447 differentially expressed genes identified in the comparison between Hy0 group and Hy6 group were significantly enriched in 46 KEGG pathways, including cellular process pathways (302 DEGs in 10 pathways), environmental information processing pathways (335 DEGs in eight pathways), and genetic information processing pathways (114 DEGs in eight pathways), human disease pathways (425 DEGs in eight pathways), metabolic pathways (74 DEGs in five pathways) and organismal systems pathways (197 DEGs in seven pathways) (Figure 5A). In addition, the top 20 enriched pathways included IL-17 signaling pathway (ko04657), HIF-1 signaling pathway (ko04066), TNF signaling pathway (ko04668), PI3K-Akt signaling pathway (ko04151), MAPK signaling pathway (ko04010), cholesterol metabolism (ko04979), protein processing in endoplasmic reticulum (ko04141) and pathways in cancer (ko05200) (Figure 5B). Overall, the top 20 pathways were mainly involved in biological processes such as inflammatory response, immune response, environmental stress, cell proliferation and differentiation, apoptosis, lipid metabolism, and protein processing. The enrichment of these pathways indicates that *E. akaara* may face oxidative stress, metabolic disorders, hormonal dysregulation, and inflammatory responses under acute hypoxia.

The 1257 differentially expressed genes in Hy0 group and Hy9 group were significantly enriched in 40 KEGG pathways, including cellular process pathways (139 DEGs in five pathways), environmental information processing pathways (173 DEGs in five pathways), genetic information processing pathways (100 DEGs in six pathways), human disease pathways (561 DEGs in 10 pathways), metabolic pathways (88 DEGs in six pathways), and organismal systems pathways (196 DEGs in eight pathways) (Figure 5C). In addition, the top 20 enriched pathways included HIF-1 signaling pathway (ko04066), cholesterol metabolism (ko04979), glucagon signaling pathway (ko04922), insulin resistance (ko04931), glycolysis/gluconeogenesis (ko00010), starch and sucrose metabolism (ko00500), arginine and proline metabolism (ko00330) (Figure 5D). Overall, the top 20 pathways were mainly involved in biologic processes such as cell metabolism, protein processing, neurodegenerative diseases, immune responses, and apoptosis.

### 3.5. RT-qPCR Verification

**Figure 6:**
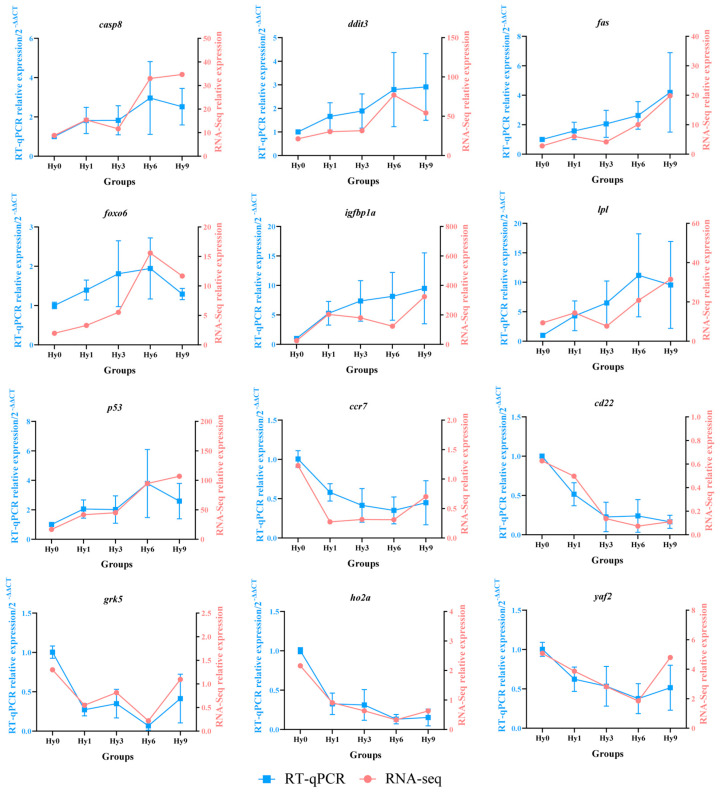
Validation of RNA-seq data by RT-qPCR analysis of 12 selected DEGs in the liver of *E. akaara* under acute hypoxic stress.

In order to verify the reliability and accuracy of RNA-seq data in the liver of *E. akaara*, several DEGs were selected for RT-qPCR verification. The relative expression levels of seven DEGs *caspase-8* (*casp8*), *DNA damage-inducible transcript 3* (*ddit3*), *fatty acid synthase* (*fas*), *forkhead box protein o6* (*foxo6*), *insulin-like growth factor-binding protein 1a* (*igfbp1a*), *lipoprotein lipase* (*lpl*) and *cellular tumor antigen* (*p53*) were significantly up-regulated under hypoxia, and the expression levels of the remaining five DEGs *C-C motif chemokine receptor 7* (*ccr7*), *B-cell receptor cd22* (*cd22*), *G protein-coupled receptor kinase 5* (*grk5*), *heme oxygenase 2a* (*ho2a*) and *YY1-associated factor 2* (*yaf2*) were significantly down-regulated (Figure 6). In brief, the results of the RT-qPCR showed similar expression trends to those based on sequencing data, supporting the reliability and accuracy of the sequencing results.

### 3.6. Key DEGs Involved in Hypoxia Responses

**Figure 7:**
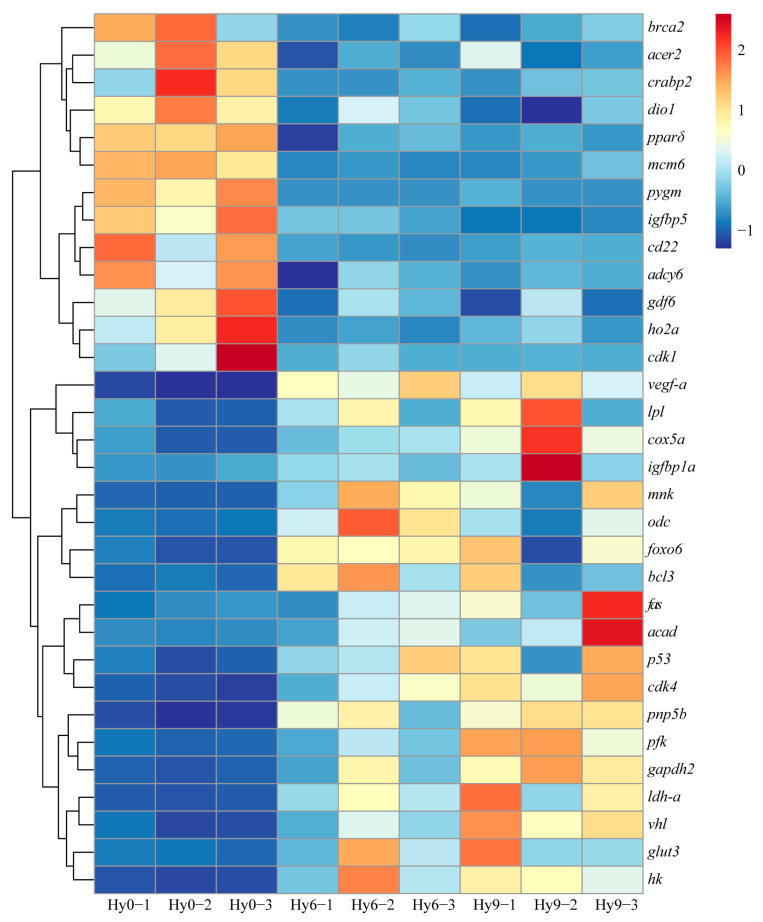
Heat map of hypoxia-responsive DEGs in the liver of *E. akaara* under acute hypoxic stress at 0 h, 6 h, 9 h. Red indicates gene up-regulated expression, blue indicates gene down-regulated expression.

On the basis of RNA-seq data, the DEGs associated with hypoxia in the liver of *E. akaara* were identified. The expression levels of these DEGs in the Hy0, Hy6, and Hy9 groups were shown in the heat map (Figure 7). Most of these DEGs were mainly involved in metabolism, immune response, oxidative stress, cell cycle regulation, cell proliferation and apoptosis. In the Hy6 and Hy9 groups, the cell-proliferation-related genes *insulin-like growth factor-binding protein 5* (*igfbp5*), *igfbp1a* and *growth/differentiation factor 6* (*gdf6*), metabolism-related genes *alkaline ceramidase 2* (*acer2*), *cellular retinoic acid-binding protein 2* (*crabp2*), *iodothyronine deiodinase 1* (*dio1*), *peroxisome proliferator-activated receptor delta* (*pparδ*) and *glycogen phosphorylase*, *muscle form* (*pygm*), cell-cycle-regulation-related genes *cyclin-dependent kinase 1* (*cdk1*) and *DNA replication licensing factor* (*mcm6*), DNA-repair-related gene *breast cancer 2* (*brca2*), signal-transduction-related gene *adenylate cyclase type 6* (*adcy6*), immune-related gene *cd22* and oxidative-stress-related gene *ho2a* were significantly down-regulated. Apoptosis-related genes *foxo6*, *p53* and *von Hippel–Lindau disease tumor suppressor* (*vhl*), immune-related genes *B-cell lymphoma 3* (*bcl3*), *MAP kinase-interacting serine/threonine-protein kinase* (*mnk*), *fas* and *purine nucleoside phosphorylase 5b* (*pnp5b*), metabolism-related genes *lpl*, *cytochrome c oxidase subunit 5a* (*cox5a*), *ornithine decarboxylase* (*odc*), *acyl-CoA desaturase* (*acad*), *phosphofructokinase* (*pfk*), *glyceraldehyde-3-phosphate dehydrogenase 2* (*gapdh2*), *lactate dehydrogenase a* (*ldh-a*), *facilitated glucose transporter member 3* (*glut3*) and *hexokinase* (*hk*), cell-cycle-regulation-related gene *cyclin-dependent kinase 4* (*cdk4*) and the angiogenesis/vascular-permeability-regulation-related gene *vascular endothelial growth factor a* (*vegf-a*) were significantly up-regulated.

### 3.7. Expression Patterns of 18 Key DEGs in Liver and Brain

**Figure 8:**
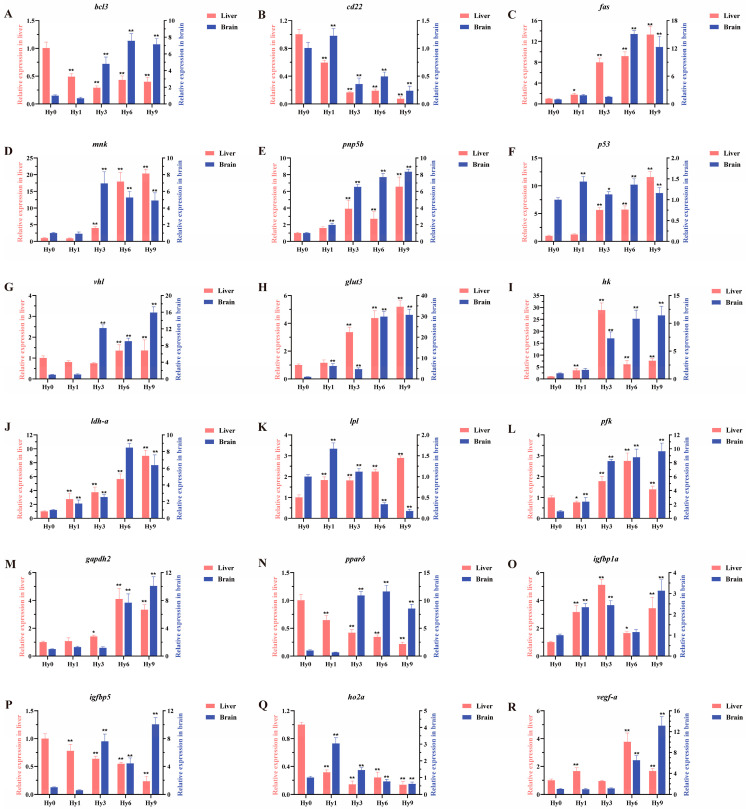
Expression analysis of 18 key DEGs in the liver (red) and brain (blue) of *E. akaara* after hypoxia stress. (**A**) *bcl3*; (**B**) *cd22*; (**C**) *fas*; (**D**) *mnk*; (**E**) *pnp5b*; (**F**) *p53*; (**G**) *vhl*; (**H**) *glut3*; (**I**) *hk*; (**J**) *ldh-a*; (**K**) *lpl*; (**L**) *pfk*; (**M**) *gapdh2*; (**N**) *pparδ*; (**O**) *igfbp1a*; (**P**) *igfbp5*; (**Q**) *ho2a*; (**R**) *vegf-a*. Asterisks indicate significant differences between the control group and hypoxia stress group at each time point. * represents significant differences (*p* < 0.05); ** represents significant differences (*p* < 0.01).

The expression levels of 18 key genes (*bcl3*, *cd22*, *fas*, *mnk*, *pnp5b*, *p53*, *vhl*, *glut3*, *hk*, *ldh-a*, *lpl*, *pfk*, *gapdh2*, *pparδ*, *igfbp1a*, *igfbp5*, *ho2a*, *vegf-a*) were examined in both the liver and brain by RT-qPCR. As shown in Figure 8, all genes exhibited significant expression of hypoxia at 6 h and 9 h. Energy-metabolism-related genes *glut3*, *hk*, *ldh-a*, *lpl*, *pfk* and *gapdh2* were basically up-regulated in the liver, and reached the peak at 9 h, 3 h, 9 h, 9 h, 6 h and 6 h, respectively. Similar up-regulated trends were observed in the brain, except for *lpl*, which was significantly down-regulated at 6 h and 9 h, and peak expression in brain occurred at 9 h, 9 h, 6 h, 1 h, 9 h and 9 h respectively (Figure 8H–M). Immune-related genes *bcl3* and *cd22* were significantly down-regulated in the liver, while other immune-related genes *fas*, *mnk* and *pnp5b* were significantly up-regulated in the liver, and all reached the peak at 9 h. In brain tissue, *cd22* was significantly up-regulated at 1 h and down-regulated at 3 h, 6 h, and 9 h, and *bcl3*, *fas*, *mnk*, and *pnp5b* were all significantly up-regulated and peaked at 6 h, 6 h, 3 h, and 9 h, respectively (Figure 8A–E). In the liver, the apoptosis-related genes *p53* and *vhl* were significantly up-regulated, and both reached the peak at 9 h. In the brain, they peaked at 1 h and 9 h, respectively (Figure 8F,G). The cell-growth- and proliferation-related genes *igfbp1a* and *igfbp5* were significantly up-regulated and down-regulated in the liver respectively. In the brain, they were all significantly up-regulated and both peaked at 9 h (Figure 8O,P). The oxidative-stress-related gene *ho2a* was significantly down-regulated in liver and was significantly up-regulated in brain at 1 h and 3 h, and peaked at 1 h, while it was significantly down-regulated at 6 h and 9 h (Figure 8Q). *Vegf-a*—a downstream gene of the key hypoxia-responsive gene *hif-1α,* and one involved in angiogenesis—was significantly expressed in both liver and brain tissues (Figure 8R). *Pparδ*, which acts as an antioxidant and maintains vascular homeostasis, was significantly down-regulated in the liver, whereas it was significantly up-regulated in brain and peaked at 6 h (Figure 8N).

### 3.8. Effects of Acute Hypoxia on Liver Enzyme Activities

**Figure 9:**
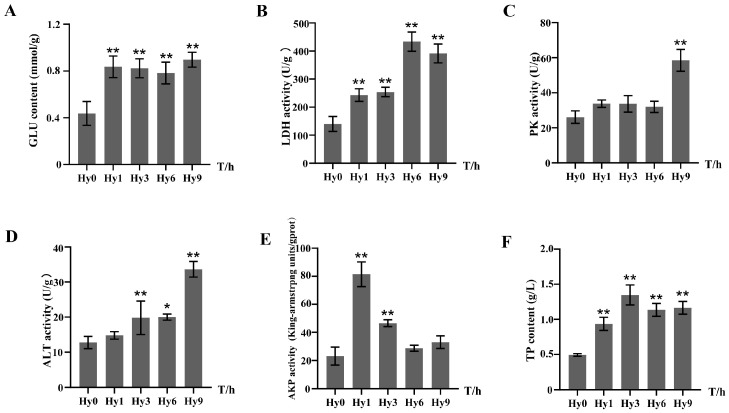
Expressions of liver indexes in *E. akaara* under hypoxia: (**A**) GLU; (**B**) LDH; (**C**) PK; (**D**) ALT; (**E**) AKP; (**F**) TP. Asterisks indicate significant differences between the control group and hypoxic stress group at each time point. * represents significant differences (*p* < 0.05); ** represents significant differences (*p* < 0.01).

To investigate the effect of acute hypoxia on the activities of enzymes related to energy metabolism in the liver tissue of *E. akaara*, we detected the content of GLU, the activity of LDH and PK. The contents (activities) of GLU and LDH in the hypoxia experimental group were significantly higher than those in the normoxia control group. GLU content peaked at 9 h, which was 2.05-fold that of the Hy0 group (Figure 9A). Peak LDH activity occurred at 6 h, at a level that was 3.09-fold higher than that in the control group (Figure 9B). PK activity did not show significant changes during the pre-acute hypoxia period, but showed a significant increase and peaked at 9 h, with a 2.24-fold difference in expression (Figure 9C). ALT and AKP were used as organism-immune-function-related enzyme activity index; ALT activity increased significantly from 3 h and reached the peak activity at 9 h, and its level was 2.64-fold that of the control group (Figure 9D). AKP activity increased significantly during the early phase of acute hypoxia at 1 h and 3 h, peaked at 1 h with a 3.52-fold difference in expression, and then returned to levels not significantly different from the control group at 6 h and 9 h (Figure 9E). Total protein (TP) content was consistently higher in the hypoxic experimental groups, peaking at 3 h at a level 2.73-fold that of the control group (Figure 9F).

## 4. Discussion

In aquaculture environments, high-density stocking, pollutant accumulation, eutrophication, and elevated temperatures frequently lead to insufficient dissolved oxygen in water [38,39]. This hypoxia environment can negatively affect the activity performance, growth and physiological functions of fish, and may lead to mass mortality of cultured fish in severe cases [40,41]. This study aimed to investigate the effects of acute hypoxia on the liver transcriptome profile and biochemical parameters of *E. akaara*. RNA-seq and RT-qPCR results revealed that signaling pathways and DEGs related to apoptosis, energy metabolism, cell cycle regulation, and immune response were significantly enriched in the Hy6 and Hy9 groups, providing new insights into the molecular mechanisms underlying hypoxia adaptation in this species at the transcriptome level.

The liver is an important target organ of fish under hypoxia stress. As the hub of metabolic regulation, the liver is widely involved in energy metabolism and the synthesis of a variety of physiological active substances [23]. In the process of maintaining homeostasis, the liver performs multiple physiological functions, including the regulation of carbohydrate, lipid, and protein metabolism, as well as essential life activities such as bile synthesis, blood formation, coagulation-factor production, and detoxification [18]. As an extremely metabolically active and highly oxygen-dependent organ, the metabolic level of the brain can reach 4 to 10 times that of the whole body, which makes it highly sensitive to hypoxic stress, and then affects the immune response, cell function and neural activity [42,43,44]. Under hypoxic stress, hypoxia-inducible factors (HIFs) act as master regulators inside the cell, which coordinate the host adaptation to hypoxic environment by activating the expression of downstream target genes [45]. Its target genes include: *igfbp1a* with growth inhibitory function [46], and *vegf-a* promoting angiogenesis [47]. Studies have shown that hypoxia can induce the expression of *igfbp1a* in zebrafish liver and *vegf-a* in the liver of *Astronotus ocellatus*, and the overexpression of *igfbp1a* in zebrafish embryos can inhibit IGF-1-induced cell proliferation, resulting in a significant reduction in growth and development rates [48,49]. Consistent with the results of the present study that *igfbp1a and vegf-a* were significantly up-regulated in the hypoxia group in *E. akaara* (Figure 8). In hypoxic environments, HIF-1 signaling regulates the expression of genes related to glucose metabolism, enabling cells to adapt to hypoxic conditions and maintain survival [50]. HIF-1 signaling pathway can activate the key genes of glucose metabolism, such as promoting the expression of glucose transporters (*glut1* and *glut3*) and enhancing the expression of glycolytic pathway enzyme genes, thereby reducing the dependence of cells on aerobic energy metabolism and relying on the anaerobic glycolysis pathway to maintain ATP supply [51,52]. In the present study, the expression levels of glucose transporter *glut3* and glycolysis-related genes *hk*, *pfk*, *ldh-a* and *gapdh2* were significantly increased in both the liver and brain under acute hypoxia stress (Figure 8), similar to the results of previous studies [53,54,55,56,57]. Glucose transporter (GLUT) is an integral membrane protein that can promote the uptake of glucose by cells through transmembrane transport, ensuring that cells can obtain necessary nutrients and maintain normal physiological functions [58,59]. On the basis of sequence homology and structural features, the GLUT family can be divided into three major subclasses, among which GLUT3 has a universal tissue distribution [60,61]. Hexosokinase (HK) is a key rate-limiting enzyme in glycolysis that phosphorylates glucose to serve as a substrate for ATP production and metabolite biosynthesis [62]. Both phosphofructokinase PFK and HK are important regulatory nodes in the glycolytic pathway, and their irreversible catalytic nature makes them play a decisive role in regulating the rate of the glycolytic pathway [63]. LDH-A is one of the key enzymes in glycolysis. It uses reduced nicotinamide adenine dinucleotide (NADH) as a coenzyme to catalyze the biological conversion of pyruvate to lactate to maintain the continuous progress of glycolysis [64,65]. Lactate generated in muscle cells enters the bloodstream and is then transported to the liver, where LDH-A reversibly converts lactate to pyruvate, which then enters the tricarboxylic acid cycle (TCA cycle) for further oxidative breakdown to generate ATP [66]. The gene expression patterns observed in the present study are consistent with the above findings, indicating that HIF-1 signaling pathway is involved in the regulation of downstream target genes and glycolytic metabolism in *E. akaara* under hypoxic conditions.

Under hypoxic stress, fish will trigger a series of physiological regulatory networks, including regulating apoptosis, inflammatory response and signal transduction, in order to maintain physiological homeostasis and improve the adaptability of the body. KEGG pathway enrichment analysis of *E. akaara* liver revealed that the DEGs related to immune response and apoptosis were significantly enriched in several key signaling pathways, including IL-17, TNF and PI3K/Akt signaling pathways (Figure 5B,D). Under hypoxic conditions, the degradation of HIF-1α is inhibited, allowing it to accumulate in the cell and translocate to the nucleus, where it subsequently interacts with the hypoxia response element (HRE) on the tumor necrosis factor-α (*tnf-α*) promoter to promote transcriptional activation of *tnf-α* [67]. TNF-α is a pro-inflammatory cytokine produced by T cells, macrophages, and other cell types, and it plays a crucial role in regulating inflammatory responses and host defense against pathogens in a variety of tissues and cell types [68]. Studies have shown that TNF-α can regulate the activity of HIF through a variety of classical cell signal transduction pathways, including nuclear factor-κB (NF-κB) and phosphatidylinositol-3-kinase/protein kinase B (PI3K/Akt) signaling pathways [69]. Activation of the PI3K/Akt signaling pathway mediated by receptor tyrosine kinases (RTKs) further up-regulates the transcriptional level of *hif-1α* [70]. *Bcl3*, *cd22*, *fas*, and *mnk* typically participate in immune regulation by maintaining immune cell homeostasis and alleviating inflammatory damage. Under hypoxic stress, *bcl3* and *cd22* were significantly down-regulated, while *fas* and *mnk* were significantly up-regulated in the liver. In the brain, *bcl3*, *fas*, and *mnk* were significantly up-regulated, while *cd22* was initially significantly up-regulated, followed by a significant down-regulation (Figure 8). Bcl3 is a classic NF-κB coactivator. In hepatocytes and liver sinusoidal endothelial cells, acute hypoxia rapidly up-regulates HIF-1α, which binds p65/RelA and blocks its nuclear translocation, thereby directly suppressing canonical NF-κB activity, and *bcl3* expression decreases accordingly [71]. CD22 is a B-cell-restricted receptor that determines the threshold for B-cell activation. Under hypoxia, hepatic macrophages secrete cytokines such as TNF-α and IL-10, and IL-10 down-regulates *cd22* at the mRNA level [72]. The significant down-regulation of *bcl3* and *cd22* in the liver implies the weakening of inflammatory signaling and the reduction in B-cell activation threshold to suppress excessive inflammation and accelerate tissue repair. Under hypoxia, the expression of *fasl* (Fas ligand) in hepatocytes increases synchronously, which induces caspase8-dependent apoptosis of pro-inflammatory cells through the Fas/FasL pathway to avoid uncontrolled inflammatory cascade and maintain immune homeostasis [73]. Hypoxia also elevates *mnk* expression, which selectively enhances translation of anti-inflammatory mRNAs such as *il-10* and *bcl-xl* while repressing translation of pro-inflammatory mRNAs including *tnf-α* and *il-1β*, thereby sustaining the liver’s inherent immune tolerant phenotypes [74]. The changes in *bcl3*, *fas*, *mnk* and *cd22* levels in the brain may reflect the complex balance between inflammation regulation, cell survival and apoptosis in neural tissue under hypoxic stress, revealing the different adaptive response mechanisms of the liver and brain to hypoxia. *P53* is a well-known pro-apoptotic tumor suppressor gene, which is maintained at a low level in normal cells. However, lethal hypoxia damage to cells will significantly up-regulate the expression of p53 and promote the apoptosis of damaged cells [75]. Under hypoxia stress, P53 recruits VHL to degrade HIF-1α, and *vhl* itself is positively regulated by *p53*. Therefore, HIF-1α is degraded after up-regulation of *vhl*, which avoids the continuous excessive glycolysis and lactic acidosis driven by HIF-1α and improves the survival rate of immune cells [57]. The significant upregulation of *p53* and *vhl* in both the liver and brain indicated their pivotal roles in eliminating damaged cells by apoptosis, prevention of metabolic runaway and maintenance of homeostasis. These findings revealed that key signaling pathways such as TNF and PI3K/Akt are activated through HIF-1α-mediated regulatory networks, coordinating inflammatory responses and apoptosis in fish under hypoxia, thereby promoting hypoxia adaptability and survival.

Fish typically enhance their ability of oxygen uptake and utilization in hypoxic waters through a series of physiological, biochemical and molecular adaptive regulation, while activating endogenous protective mechanisms to reduce physiological damage caused by hypoxia [76]. However, when the compensatory mechanism of maximizing oxygen uptake fails to effectively maintain oxygen homeostasis, individuals will change the way of energy metabolism and inhibit the consumption of energy metabolism through other physiological and biochemical reactions [77]. During anaerobic metabolism, glucose (GLU) acts as a key metabolic substrate, decomposing to pyruvate and a small amount of ATP, while lactate dehydrogenase (LDH) catalyzes the reduction in pyruvate to lactate in the glycolytic pathway to maintain energy supply in the absence of oxygen [78,79]. In the present study, hypoxia stress significantly increased liver GLU content and LDH activity in *E. akaara* (Figure 9), and similar results were found in *Larimichthys polyactis* [80]. Pyruvate kinase (PK) plays a key role in glycolysis, which is the main energy supply pathway of tissues. PK catalyzes the irreversible transphosphorylation of phosphoenolpyruvate (PEP) and ADP to generate pyruvate and ATP [81,82]. In the present study, PK activity did not change significantly in the early stages of hypoxia, but increased significantly as exposure continued (Figure 9), indicating that the anaerobic metabolism of *E. akaara* was enhanced under hypoxic conditions [83,84].

Hypoxia stress induces excessive production of reactive oxygen species (ROS), triggering oxidative stress in organisms, causing oxidative damage and inflammation, and ultimately disrupting their normal physiological functions [85]. Elevated intracellular ROS levels lead to lipid peroxidation, increasing hepatocyte membrane permeability and thereby promoting the release of liver enzymes such as alanine aminotransferase (ALT) into the extracellular space [86]. ALT catalyzes the transfer of amino groups from alanine to α-ketoglutarate to form pyruvate and glutamate, and pyruvate is the substrate of gluconeogenesis. Increased ALT activity may indicate that the organism needs to enhance glucose synthesis through the gluconeogenic pathway to adjust the pattern of energy metabolism [87]. In addition, when liver cells are damaged by toxic substances and external environmental stress, it may lead to ALT leakage from cells into interstitial fluid. Therefore, ALT is commonly regarded as a biomarker of liver injury [88,89]. In this study, acute hypoxia stress significantly increased the liver ALT activity of *E. akaara* (Figure 9); similar results have also been reported in black rockfish (*Sebastes schlegelii*) [90]. Alkaline phosphatase (AKP) is not only a key regulatory enzyme for transferring phosphate groups, but also a multifunctional enzyme that widely participates in organism metabolism and innate immune response. Changes in AKP activity may indirectly reflect the immune level of fish [91]. There is a positive correlation between AKP activity and the level of stress in the organism. The increase in AKP activity can be used as an important biochemical index to evaluate the organism under adverse environmental stress [92,93,94]. In this study, we found that AKP activity in the liver of *E. akaara* was significantly increased during the early stage of acute hypoxia and then returned to the basal level (Figure 9). Similarly, it was found that AKP activity in the liver of hybrid yellow catfish (*Pelteobagrus fulvidraco* × *P. vachelli*) was significantly increased under 1.5 mg/L dissolved oxygen hypoxia and then returned to normal levels [92]. These results indicate that hypoxia induces oxidative stress in the liver, which in turn initiates immune defense to maintain cellular homeostasis.

## 5. Conclusions

In this study, we analyzed the effects of acute hypoxia on RNA expression profiles and biochemical parameters in *E. akaara* using a combination of RNA-seq, RT-qPCR, and physiological and biochemical assays. RNA-seq results of liver samples showed that more DEGs were identified in 6 h and 9 h, with 2165 and 1833 DEGs, respectively. These DEGs were mainly enriched in pathways related to energy metabolism, immune response and apoptosis, including IL-17, HIF-1, TNF and PI3K/Akt signaling pathways. RT-qPCR revealed that genes related to energy metabolism (*glut3*, *hk*, *ldh-a*, *lpl*, *pfk*, *gapdh2*), immune response (*bcl3*, *cd22*, *fas*, *mnk*, *pnp5b*) and apoptosis (*p53*, *vhl*) were significantly changed in the liver and brain at 6 h and 9 h under acute hypoxia. In addition, the activities of the hepatic glycolytic pathway and immune-related enzymes were changed significantly at 6 h and 9 h after acute hypoxia. These results indicate that 6 h and 9 h are the critical window period for the physiological stress responses in *E. akaara*. These physiological responses together reflect the adaptive regulation of the organism to maintain homeostasis under hypoxia stress. This study would help to understand the response of *E. akaara* to hypoxia stress, and provide direct theoretical basis and candidate indicators for the breeding of *E. akaara* for hypoxia tolerance.

## Figures and Tables

**Table 1:** Specific primers for RT-qPCR.

Gene	Primer Sequence (5′ → 3′)	Amplicon Size (bp)	Reference
*casp8*	F: CACGTGGTTTGTGTGTGGTC R: CAAGCCCAGCTTTTGCAGTT	185	#
*ddit3*	F: CCGGCTGGGAAAACGTAAGA R: CTGCTCTCGTTCTTTGCGAC	130	#
*fas*	F: ATCAACAGACTGCAGCGGAA R: CCATTTAGCTCCAGGCCACA	155	#
*foxo6*	F: CCTGAGGATGGACTGTTGGG R: AATGTGAGGCCGTCGATCAG	101	#
*igfbp1a*	F: CAACTGTGACAAACACGGGC R: GGCAAGTCGGTCGAACCTAA	126	#
*lpl*	F: ACACCCTGTCCAAAGACGAC R: TTGATGCCTTCCAGTGCGAT	187	#
*p53*	F: GATGGTGTTACTCCCCCGTC R: TTCAATGGGGGTGGTCTTGG	171	#
*ccr7*	F: ATCAGCGTGGAACGCTACTT R: ACACGGAGCTTGTCAGCATT	200	#
*cd22*	F: ACCAACCGCATCAGTACAGG R: CGTTGACAGTGGGAAGCTCT	153	#
*grk5*	F: TCGTCGAGCCTTCGTTCAAA R: GCTGCCTGTGTTGAACTTGG	140	#
*ho2a*	F: TGAGATGTTAGCCGAGGGGA R: GATTGGGGCGATGTCAGGAT	190	#
*yaf2*	F: GACGAGGGGTTTTGGGACTG R: GGACGAGGCTTCCTTGTTGA	110	#
*bcl3*	F: GCTGAAAACCTGCTGACTGC R: GTGGAGTCTGACGGAGGTTG	175	#
*mnk*	F: GCCCAAGCTCTTGACTTCCT R: CACTGCTGAGCTTCACTCCA	145	#
*pnp5b*	F: TGATGAGAGGTTTGGCGTCC R: AAGTCGCTGTAGCCCAGTTC	105	#
*vhl*	F: CAGCCTCTTCCTCTGGTTCG R: GTAGGTCCACGTATGCTCGG	133	#
*glut3*	F: GCTCCCCAAACTACCGTCAA R: CAGCTCCTATGGTGGCGTAG	150	#
*hk*	F: GTGACAGGTTGGCTTTGCTG R: CATTCCTGCCCCGCATATCT	140	#
*ldh-a*	F: GATCATCGGAGAGCATGGGG R: GCCCTTCAGCTTGATGACCT	175	#
*pfk*	F: AGCCATCGACAGAAATGGCA R: CTGTCAAACGCTGAGGGAGT	135	#
*gapdh2*	F: GTGCCCACGCAAACATCATT R: AGATGCAGGCTTGGACAGAC	161	#
*pparδ*	F: AGCCAGTTAGTTCCTGGTGC R: CAAACCCCGGAATGCACTTG	127	#
*igfbp5*	F: CAAGCAGTGCAAGCCATCTC R: GTTGCTGCTCTCCAGGTCTT	130	#
*vegf-a*	F: CCGGAGGACACTGAACACAA R: GACATTGCGAGTTTCCGTGG	111	#
*β-actin*	F: AAGGACCTGTACGCCAACAC R: AATCCACATCTGCTGGAAGG	#	[36]
*ef-1β*	F: CCACATCAAATCCTACCAGAGCCAGA R: GTCGTCGTCCTCCTCGTCATCTTT	#	[37]

# The primers were designed by Primer v.6.0 software.

**Table 2:** Summary of RNA-seq data of *Epinephelus akaara*.

Sample	Raw Reads	Clean Reads	Clean Bases (GB)	Q20%	Q30%	GC%	Total Mapped Rate (%)
Hy0-1	40,305,086	38,886,524	5.83 G	99.45	96.77	49.50	91.54
Hy0-2	36,980,632	35,775,540	5.37 G	99.44	96.60	49.50	91.90
Hy0-3	36,687,824	35,569,460	5.34 G	99.47	96.60	49.50	90.68
Hy1-1	38,324,276	36,942,978	5.54 G	99.42	96.15	51.00	92.13
Hy1-2	39,083,626	37,920,954	5.69 G	99.41	96.37	50.50	92.86
Hy1-3	39,412,782	38,103,166	5.72 G	99.41	96.73	49.50	93.46
Hy3-1	43,238,602	41,836,390	6.28 G	99.55	97.02	49.50	94.01
Hy3-2	33,847,454	32,608,120	4.89 G	99.43	96.63	49.50	93.52
Hy3-3	41,283,758	40,158,204	6.02 G	99.45	96.94	54.50	93.82
Hy6-1	41,776,942	40,621,678	6.09 G	99.47	96.83	52.50	93.29
Hy6-2	40,154,980	38,896,146	5.83 G	99.49	96.62	49.50	93.03
Hy6-3	41,499,992	40,281,998	6.04 G	99.49	96.84	49.50	93.72
Hy9-1	37,295,278	36,127,082	5.42 G	99.47	96.65	49.50	93.36
Hy9-2	43,946,036	42,439,418	6.37 G	99.48	96.95	48.50	91.04
Hy9-3	40,743,352	39,310,352	5.90 G	99.44	96.89	49.50	93.45

## Data Availability

Data will be made available on request.
